# Land Use Planning and Wildfire: Development Policies Influence Future Probability of Housing Loss

**DOI:** 10.1371/journal.pone.0071708

**Published:** 2013-08-14

**Authors:** Alexandra D. Syphard, Avi Bar Massada, Van Butsic, Jon E. Keeley

**Affiliations:** 1 Conservation Biology Institute, La Mesa, California, United States of America; 2 Department of Biology and Environment, University of Haifa at Oranim, Kiryat Tivon, Israel; 3 Humboldt University-Berlin, Berlin, Germany; 4 U.S. Geological Survey, Western Ecological Research Center, Sequoia – Kings Canyon Field Station, Three Rivers, California, United States of America; 5 University of California, Los Angeles, California, United States of America; DOE Pacific Northwest National Laboratory, United States of America

## Abstract

Increasing numbers of homes are being destroyed by wildfire in the wildland-urban interface. With projections of climate change and housing growth potentially exacerbating the threat of wildfire to homes and property, effective fire-risk reduction alternatives are needed as part of a comprehensive fire management plan. Land use planning represents a shift in traditional thinking from trying to eliminate wildfires, or even increasing resilience to them, toward avoiding exposure to them through the informed placement of new residential structures. For land use planning to be effective, it needs to be based on solid understanding of where and how to locate and arrange new homes. We simulated three scenarios of future residential development and projected landscape-level wildfire risk to residential structures in a rapidly urbanizing, fire-prone region in southern California. We based all future development on an econometric subdivision model, but we varied the emphasis of subdivision decision-making based on three broad and common growth types: infill, expansion, and leapfrog. Simulation results showed that decision-making based on these growth types, when applied locally for subdivision of individual parcels, produced substantial landscape-level differences in pattern, location, and extent of development. These differences in development, in turn, affected the area and proportion of structures at risk from burning in wildfires. Scenarios with lower housing density and larger numbers of small, isolated clusters of development, i.e., resulting from leapfrog development, were generally predicted to have the highest predicted fire risk to the largest proportion of structures in the study area, and infill development was predicted to have the lowest risk. These results suggest that land use planning should be considered an important component to fire risk management and that consistently applied policies based on residential pattern may provide substantial benefits for future risk reduction.

## Introduction

The recognition that homes are vulnerable to wildfire in the wildland-urban interface (WUI) has been established for decades [e.g., 1,2]; but with a recent surge in structures burning, this issue is now receiving widespread attention in policy, the media, and the scientific literature. Single fire events, like those in Greece, Australia, southern California, and Colorado have resulted in scores of lost lives, thousands of structures burned, and billions of dollars in expenditures [3]–[6]. With the potential for increasingly severe fire conditions under climate change [7] and projections of continued housing development [8], it is becoming clear that more effective fire-risk reduction solutions are needed. “Fire risk” here refers to the probability of a structure burning in a wildfire within a given time period.

Traditional fire-risk reduction focuses heavily on fire suppression and manipulation of wildland vegetation to reduce hazardous fuels [9]. Enormous resources are invested in vegetation management [10], but as increasing numbers of homes burn down despite this massive investment, the “business-as-usual” approach to fire management is undergoing reevaluation. One issue is that fuel treatments may not be located in the most strategic positions, i.e., in the wildland-urban interface [11]. Yet, even if treatments surrounded all communities, scattered development patterns are difficult for firefighters to reach [12]–[14], and fuel treatments do little to protect homes without firefighter access [15]–[16]. Fuel treatments may also be ineffective against embers or flaming materials that blow ahead of the fire front [17].

One alternative to traditional fire management that is receiving widespread attention is to prepare communities through the use of fire-safe building materials or creating defensible space around structures [17]–[18]. These actions represent an important shift in emphasis from trying to prevent wildfires in fire-prone areas to better anticipating fires that are ultimately inevitable. Nevertheless, the cost of building and retrofitting homes to be fire-safe can be prohibitive, and these actions do not guarantee immunity from fire [19].

Land use planning is an alternative that represents a further shift in thinking, beyond the preparation of communities to withstand an inevitable fire, to preventing new residential structures from being exposed to fire in the first place. The reason homes are vulnerable to fires at the wildland-urban interface is a function of its very definition: “where homes meet or intermingle with wildland vegetation” [20]. In other words, the location and pattern of homes influence their fire risk, and past land-use decision-making has allowed homes to be constructed in highly flammable areas [21]. Land use planning for fire safety is beginning to receive some attention in the literature [22]–[23], and there is growing recognition of the potential benefits of directing development outside of the most hazardous locations [8], [19], [24].

Despite recent attention in the literature, land use planning for wildfire has yet to gain traction in practice, particularly in the United States. However, fire history has been used to help define land zoning for fire planning in Italy [22], and bushfire hazard maps are integrated into planning policy in Victoria, Australia [25]. Although some inertia inevitably arises from complications with existing policy and plans, a primary impediment to the design and implementation of fire-smart land use planning is lack of guidance about specific locations, patterns of development, or appropriate methodology to direct the placement of new development. Without a solid knowledge base to draw from, planners will be misinformed about which planning decisions may result in the greatest overall reduction of residential landscape risk. Even worse, poor science could result in placement of homes in areas that actually have high fire hazard.

Research on how planning decisions contributed to structures burning in the past provides some guidance about what actions may work in the future. Analysis of hundreds of homes that burned in southern California the last decade showed that housing arrangement and location strongly influence fire risk, particularly through housing density and spacing, location along the perimeter of development, slope, and fire history [26]. Although high-density structure-to-structure loss can occur [27]–[28], structures in areas with low- to intermediate- housing density were most likely to burn, potentially due to intermingling with wildland vegetation or difficulty of firefighter access. Fire frequency also tends to be highest at low to intermediate housing density, at least in regions where humans are the primary cause of ignitions [29]–[30].

These results suggest, for example, that placing new residential development within the boundaries of existing high-density developments or in areas of low relief may reduce fire risk. However, it is difficult to know whether broad-scale planning policies would actually result in the intended housing arrangement and pattern at the landscape scale, and whether those patterns would result in lower fire risk. Our objective here was to simulate three scenarios of future residential development, and to project wildfire risk, in a rapidly urbanizing and fire-prone region where we have studied past structure loss [25]. We based all future development on an econometric subdivision model, but we varied the emphasis of subdivision decision-making based on three broad and common growth types.

Although cities vary in extent, fragmentation, and residential density [31]–[32], urban form typically adheres to a set of common patterns [33]–[34], and we based our development scenarios on the three primary means by which residential development typically occurs: infill, expansion, or leapfrog [34]. Infill is characterized by development of vacant land surrounded by existing development, typically in built-up areas where public facilities already exist. [35]–[36], and should result in higher structure density rather than increased urban extent. Expansion growth occurs along the edge of existing development, extends the size of the urban patch to which it is adjacent, and may have variable influence on structure density. Leapfrog growth occurs when development occurs beyond existing urban areas such that the new structure is surrounded by undeveloped land. This type of growth would expand the urban extent and initially result in lower structure density; but these areas may eventually become centers of growth from which infill or expansion can occur. We asked:

1. Do residential development policies reflecting broad growth types affect the resulting pattern and footprint of development across the landscape?
2. Do differences in extent, location, and pattern of residential development translate into differences in wildfire risk, based on the current configuration of structures?
3. Which development process, infill, expansion, or leapfrog, results in the lowest projected fire risk across the landscape?

## Methods

### Study Area

The study area included all land within the South Coast Ecoregion of San Diego County, California, US, encompassing an area of 8312 km^2^. The region is topographically diverse with high levels of biodiversity, and urban development has been the primary cause of natural habitat loss and species extinction [37]. Owing to the Mediterranean climate, with mild, wet winters and long summer droughts, the native shrublands dominating the landscape are extremely fire-prone. San Diego County was the site of major wildfire losses in 2003 and 2007 [38], although large wildfire events have occurred in the county since record-keeping began, and are expected to continue, as fire frequency has steadily increased in recent decades [29], [39]. The county is home to more than three million residents, and approximately one million more people are expected by 2030 [40]. Although most residential development has been concentrated along the coast, expansion of housing is expected in the eastern, unincorporated part of the county.

### Econometric Subdivision Model

A host of alternative modeling approaches exist to simulate future land use scenarios [41], including a cellular automaton model that we previously applied to the study area [42]. We chose to use an econometric modelling approach for this study because we wanted to capture fine-scale, structure-level patterns and processes that are correlated with housing loss to wildfire [26]; and econometric models may perform better at the scale of individual parcels [43].

Although we based the three development scenarios on generalized planning policies, we also wanted to ensure that the residential projections were realistic and adhered to current planning regulations. The objective of the econometric modeling was to estimate the likelihood that residential parcels will subdivide in the future. Therefore, we used a probit model to estimate the transition probability of each parcel based on a range of potential explanatory variables typically associated with parcel subdivision and housing development [44]–[45].

To develop the model of subdivision probability, we acquired GIS data of the county’s parcel boundaries in years 2005 and 2009 from the San Diego Association of Governments (SANDAG). The dependent variable was equal to 1 if a parcel subdivided between 2005 and 2009, and zero otherwise. Using these data layers we first determined which parcels were legally able to subdivide given current land use regulations. Minimum lot size restrictions are typically considered the most import restriction for determining future land use. We deemed a parcel eligible for subdivision if the current lot size was greater than twice the minimum legal size given the land class. To determine which parcels subdivided between 2005 and 2009, we queried parcel IDs where the total area was reduced by at least the minimum lot size between the two time periods. Finally, we were able to generate a suite of variables that determine the likelihood of a parcel developing in the future (Table S1).

We overlaid the parcel boundaries over a range of GIS layers representing our explanatory variables. These data are available to download at (http://www.sandag.org/index.asp?subclassid=100&fuseaction=home.subclasshome). Our explanatory variables included: parcel size, parcel size squared, six dummy variables which capture non-linear effects of parcel size, distance to the coast, distance to the coast squared; distance to city center and its square, current zoning, slope, land use, roads, if the parcel is in a protected area, if the parcel is in a development area, if the parcel is in the redevelopment area (Table 1).

**Table 1:** Variables and results from the probit regression model of parcel subdivision in San Diego County.

Subdivided (1 = yes,0 = no)	Coefficient	Std. Err.	z	P>|z|	[95% Conf. Interval]	
**Acres of lot**	0.0026342	0.00075	3.51	0	0.001164	0.004105
**Acres of lot ^2^**	−3.02E-06	1.29E-06	−2.34	0.019	−5.55E-06	−4.93E-07
**Distance to ocean**	−7.42E-06	1.33E-06	−5.59	0	−0.00001	−4.82E-06
**Distance to ocean ^2^**	2.33E-11	8.28E-12	2.82	0.005	7.11E-12	3.96E-11
**Distance to major road**	2.17E-07	2.74E-06	0.08	0.937	−5.16E-06	5.59E-06
**Distance to major road ^2^**	−1.94E-11	1.70E-11	−1.14	0.252	−5.27E-11	1.38E-11
**Distance to nearest city center**	−0.0000115	1.70E-06	−6.76	0	−1.5E-05	−8.16E-06
**Distance to nearest city center ^2^**	2.89E-11	9.70E-12	2.98	0.003	9.91E-12	4.79E-11
**Slope between 0–5%**	0.6211289	0.211761	2.93	0.003	0.206085	1.036173
**Slope between 5–10%**	0.3911427	0.210684	1.86	0.063	−0.02179	0.804076
**Slope between 10–25%**	0.0716669	0.212725	0.34	0.736	−0.34527	0.4886
**Rural Residential**	−0.3563149	0.071512	−4.98	0	−0.49648	−0.21615
**Single Family**	0.1361149	0.068678	1.98	0.047	0.001509	0.270721
**Multi-Family**	−0.2505093	0.151486	−1.65	0.098	−0.54742	0.046397
**Road**	0.015329	0.086094	0.18	0.859	−0.15341	0.184069
**Open Space**	−0.7440933	0.099145	−7.51	0	−0.93841	−0.54977
**Orchard/Vineyard**	−0.5813305	0.097867	−5.94	0	−0.77315	−0.38951
**Agriculture**	−0.9785208	0.132734	−7.37	0	−1.23867	−0.71837
**Vacant Land**	−0.5222501	0.074586	−7	0	−0.66844	−0.37606
**Zoned protected**	0.253769	0.076881	3.3	0.001	0.103086	0.404452
**Area marked for redevelopment**	−0.2680261	0.14069	−1.91	0.057	−0.54377	0.007722
**Area marked for development**	0.5780101	0.064103	9.02	0	0.452371	0.703649
**Parcel between 10–20 acres**	−0.3379532	0.065899	−5.13	0	−0.46711	−0.20879
**Parcel between 5–10 acres**	−0.6119036	0.067012	−9.13	0	−0.74325	−0.48056
**Parcel between 2–5 acres**	−1.16297	0.07062	−16.47	0	−1.30138	−1.02456
**Parcel between 1–2 acres**	−1.563956	0.090286	−17.32	0	−1.74091	−1.387
**Parcel between.5–1 acres**	−1.999939	0.099893	−20.02	0	−2.19573	−1.80415
**Parcel between.25–.5 acres**	−2.178273	0.117101	−18.6	0	−2.40779	−1.94876
**Constant**	−1.397931	0.227467	−6.15	0	−1.84376	−0.9521

Sample size 113 001, LR Chi^2^ 1535.23, pro>chi 0, pseudo R^2^ 0.22. Further description of the variables is provided in Table S1.

### Spatial Model of Future Development under Planning Alternatives

The outcome of the land use change econometric model is the subdivision probability for each parcel for a five-year time step. Based on these probabilities, we developed a GIS spatial simulation model of future land use under three distinct planning scenarios: infill (development in open or low density parcels within already developed areas), expansion (development on the fringe of developed areas), and leapfrog (development in open areas). The model runs in four 5-year time steps from 2010 to 2030, and generates the spatial locations of new housing units in the county.

Although development decisions could feasibly depend on fire risk, we did not model that here. There is no evidence that fire has influenced past regional planning decisions, so it was not used as an explanatory variable in the econometric model. Although we could have evaluated the potential for future development decisions to be based in part on fire risk, this would have required simulation of feedbacks between fires and probability of development. Because our objective in this study was to isolate the effects of the three distinct growth types, we modeled fire risk only as a function of development pattern and not vice versa.

We constructed a complete spatial database of existing residential structures in the study area [26]. These structures and their corresponding parcel boundaries served as the initial conditions for all three scenarios of the spatial simulation model. The current and projected future GIS layers of structures were also subsequently used in the fire risk model (see below). The dataset of existing housing includes locations of 687,869 structures, of which 4% were located within the perimeter of one of 40 fires that burned since 2001. During these fires, 4315 structures were completely destroyed, and another 935 were damaged.

For future development scenarios, we wanted to allocate an equal number of new structures to the landscape. This was to ensure that any predicted difference in fire risk was a function of the arrangement and location of structures, not the total number of structures. Nevertheless, differences in the total number of structures were simulated with each of the 5-year time steps. We determined the number of housing units to add during the simulations based on projections made by San Diego County [46]. Using factors such as development proposals, general plan densities, and information from jurisdictions, the county estimated that between 331,378 units and 486,336 units could be supported within the developable residential land by 2030. Because the eastern, desert portion of the county was not included in our study area, we used a conservative approach and simulated the addition of 331,378 new dwelling units. We divided this number by four to define the number of new dwelling units to add at each time step, assuming a linear growth rate.

One output of the econometric model was the prediction of the maximum number of new dwelling units that could be added to each parcel. However, dwelling units may consist of apartments as well as single family homes. The mix of single and multifamily units in the region has remained relatively constant over time, and the overall trend has been a mix of roughly 1/3 multifamily and 2/3 single family units. Because the fire risk model is based on points representing structure locations across the landscape, regardless of the number of dwelling units per structure, we needed to generate a conversion factor from dwelling units to structures. We therefore defined a minimum lot size of 0.25 acre on which no more than a single structure could be built, regardless of the number of dwelling units in it (i.e., a single family home or apartment complex). Then, once a parcel was selected for development by the model (see details below), we divided its total area by the maximum number of dwelling units to be added, according to the econometric model. If the result was larger than 0.25, we subdivided parcels according to the result. If not, we quantified how many 0.25 acre parcels fit into the original parcel, and generated the new parcel boundaries accordingly.

Using the initial map of parcels (year 2010), we classified each parcel that was defined as eligible for development (in the previous stage) as suitable for one of the three planning scenarios described above, according to the number of developed parcels in its immediate neighborhood (i.e., those parcels that share a boundary with the focal parcel). We defined ‘developed parcels’ as ones that had more than one house per 20 acres (8.09 ha). Therefore, according to these density thresholds, we allowed some parcels with nonzero housing density to be considered as ‘undeveloped’ because these large, rural parcels might contain a single or a handful of houses but they exist within a large open area. In other words, the overall land cover of these parcels was effectively undeveloped, and we therefore assumed that development in adjacent parcels would be akin to development in open areas.

We defined infill parcels as those that were completely surrounded by developed parcels. Expansion parcels had at least one neighboring parcel that was undeveloped; and leapfrog parcels were those with no developed parcels in their immediate surroundings. We reclassified the type of each available parcel in the same manner after each time step, to account for changing dynamics in the development map of the county.

We conducted three simulations, one for each development scenario (infill, expansion, and leapfrog). In each simulation, all parcels were eligible to subdivide, regardless of their class. Therefore, to build a simulation for a specific scenario, we increased the development probability of parcels of the selected scenario by 20%, to favor their development compared to the other types of parcels, without prohibiting development in the other parcel types. This approach was necessary because the projected number of dwelling units was much larger than it would be possible to fit in infill and leapfrog class parcels solely. For example, as the spatial coverage of developed parcel expands, there is less contiguous area that is undevelopable and suitable for leapfrog development. Therefore, the scenarios are not exclusive, but rather a mixture of the three development types. Yet, in each scenario, there is one main type of development, and smaller amounts of development events of the other two types.

Due to the immense computational demand of the simulations, we adopted a deterministic, rather than a stochastic approach to decide on which parcels were subdivided. After enhancing the transition probability according to the corresponding scenario, we ranked and then sorted all parcels according to their probability of subdivision. We then sequentially selected parcels, while simultaneously tallying the number of dwelling units in them, until the development target in that time step (one fourth of the total number of dwelling units to be added: 82,795) was reached. Once the development target was reached, we moved to the next time step. After each time step, the remaining parcels that were still eligible for development were re-classified to development types according to the new spatial configuration of the landscape.

Once a parcel was selected for subdivision, and the number of new parcels to develop in it was calculated (as detailed above), an equal-area spatial splitting model was employed to split the parent parcel to the predefined number of equal-area child parcels. We developed a simple splitting model which is based on iterative splitting of larger parcels into two smaller parcels using a straight line splitting boundary. Once the parcel was fully split into the needed number of sub-parcels, we allocated a new structure inside each new parcel by generating a point at its centroid (center of gravity). The point datasets of all structure locations per time step per scenario were passed over to the fire risk model, which is described below.

### Fire Risk Modeling and Analysis

To project the distribution of fire risk under alternative scenarios, we used MaxEnt [47]–[48], a map-based modeling software used primarily for species distribution modeling [48], but we have used it successfully for ignition modeling [50] and for projecting current fire risk in the study area [26]. For this study, we slightly modified the model from Syphard et al. [26]. The dependent variable was the location of structures destroyed by fire between 2001 and 2010. Although inclusion of damaged structures in the data set does not significantly affect results [26], we only included completely destroyed structures to avoid the introduction of any uncertainty.

The MaxEnt software uses a machine-learning algorithm that iteratively evaluates contrasts among values of predictor values at locations where structures burned versus values distributed across the entire study area. The model assumes that the best approximation of an unknown distribution (i.e., structure destruction) is the one with maximum entropy. The output is an exponential function that assigns a probability to every cell of a map. Thus, the resulting continuous maps of fire risk represented the probability of a structure being destroyed by fire. In these output maps, areas of predicted high fire risk that did not have structures on them represented environmental conditions similar to those in which structures have actually burned.

We based the explanatory variables on those that were significantly related to burned structures in Syphard et al. [26], including maps depicting housing arrangement and pattern, housing location, and biophysical factors. Housing pattern variables reflected individual structure locations as well as the arrangement of structures within housing clusters. We calculated housing clusters, defined as groups of structures located within a maximum of 100 m from each other, by creating 100 m buffers around all structures and dissolving the overlapping boundaries [51].

Because burned structures were significantly related to small housing clusters [26], we calculated the area of every cluster as an attribute, and then created raster grids based on that attribute. Low-to intermediate housing density and distance to the edge of the cluster were also significant explanatory variables relative to housing pattern and location [26], so we also created raster grids for those. GIS buffer measures at 1-km have been found to explain approximately 90% of the variation in rural residential density [52], so we developed density grids using simple density interpolation based on a 1-km search radius, with area determined through square map units. To create grids representing distance to the edge of clusters, we first collapsed the cluster polygons into vector polyline files, and then created grids of interpolated Euclidean Distance to the edge within each cluster.

Because the MaxEnt model randomly selects background samples in the map to compare with locations of destroyed structures, we used a mask to restrict sampling to the developed environment within cluster boundaries; the distance to the edge of the cluster would represent a different relationship inside a cluster boundary versus outside in the wildland. We also modified the grids to ensure that any random sample located within the 100m buffer zone would receive a value of 100m; thus, all points within the buffer were considered “the edge of the development”.

After creating the grids representing housing pattern and arrangement of the current configuration of structures, we applied the same algorithms to the maps of simulated future structure locations. We thus generated grids representing future housing pattern and arrangement under alternative development scenarios. The other explanatory variables, including fire history, slope, fuel type, southwest aspect, and distance to coast [26] remained constant through time for current and future scenarios. Although historic fire frequency and fuel type typically change through time, we did not simulate their dynamics here because we wanted to isolate the effect of planning decisions on housing pattern and arrangement while holding everything else constant.

We conditioned the MaxEnt model on present distributions of housing using ten thousand random background points and destroyed structures located no closer than 500-m to minimize any effect of spatial autocorrelation. We used 80% (260 records) of these data for model training, and 20% [66 records) for testing. We repeated the process using cross-validation with five replicates and used the average of these five models for analyses. For smoother functions of the explanatory variables, we used hinge features, linear, and quadratic with an increase in regularization of beta set at 2.5, based on Elith et al. [48]. The smoother response curves minimize over fitting of the model. We conducted jackknife tests of explanatory variable importance.

We first developed the model using mapped explanatory variables derived from the current configuration of structures. To project fire risk under the different time steps of the alternative development scenarios, projected the model conditioned upon current conditions onto maps representing future conditions by substituting the grids representing future housing pattern and arrangement. This is similar to how potential future distributions of species are projected under climate change scenarios [49].

To quantify differences among current and future alternative scenarios, we calculated metrics representing housing density, pattern, and footprint to determine the extent to which the planning policies produced differences in housing pattern and location. We compared the modeled structure fire risk of the scenarios by overlaying all maps of structure locations with their respective mapped output grids from the MaxEnt models and calculating probability of burning for every structure point. We also calculated total area of risk by selecting three threshold criteria [53]. These criteria, at 0.05, 0.25, and 0.5 represented three different degrees of risk, and we calculated the proportion of structures that were located in risk areas for every time step in all scenarios.

## Results

The probit econometric model, run on 113 001 observations, showed that larger parcels were most likely to subdivide, although the relationship between parcel size and subdivision probability was non-linear (Table 1). Parcels closer to existing roads, the ocean, those with lower slopes, and those designated as fit for development were all most likely to develop. Parcels designated in redevelopment areas were less likely to develop. Overall, the model had a pseudo r –squared of 0.22.

**Figure 1:**
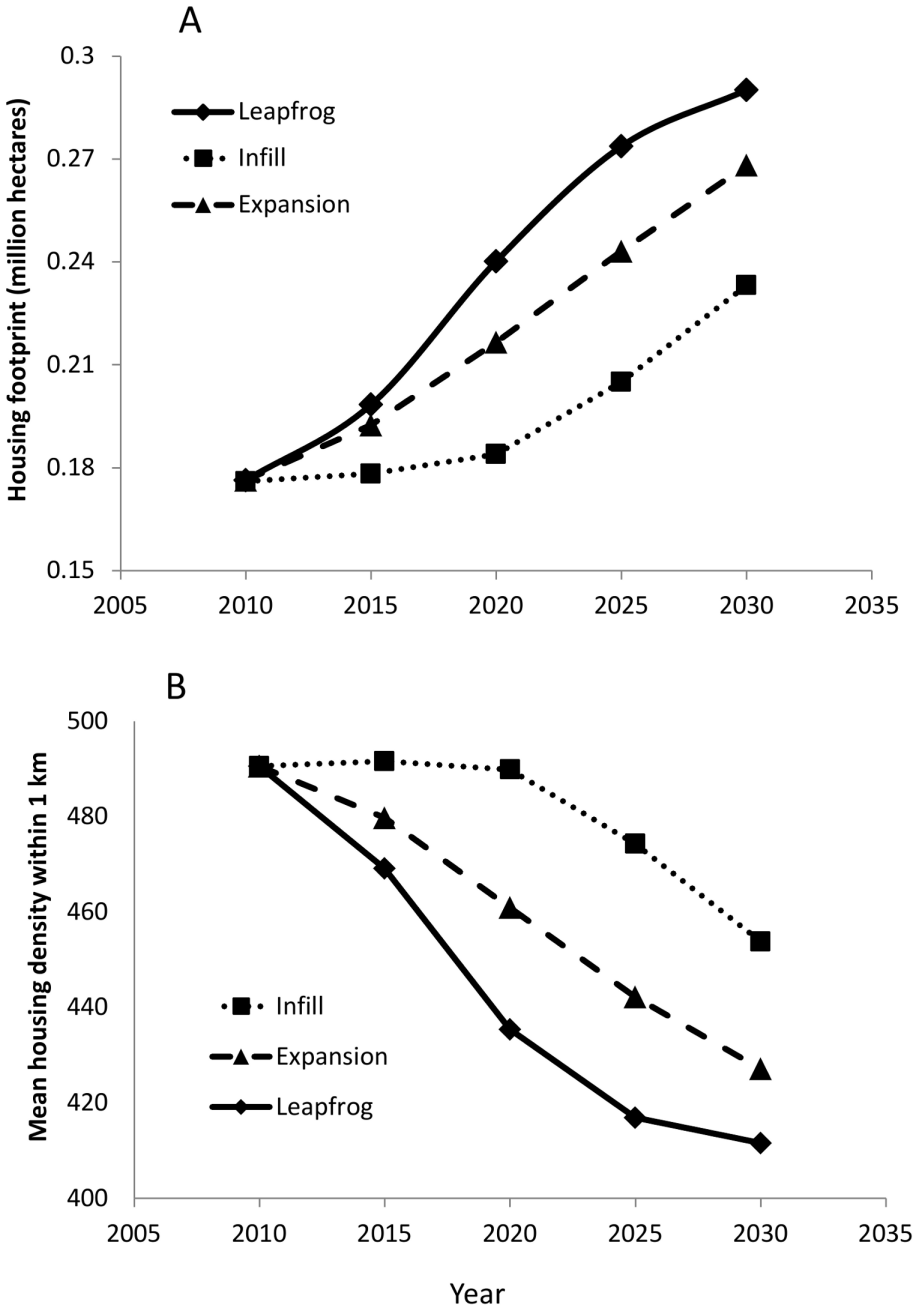
Trends of development extent and pattern for three planning policy simulations from 2010–2030, including A) total housing footprint representing the area of land within all housing clusters, and B) mean housing density averaged across all housing clusters.

**Figure 2:**
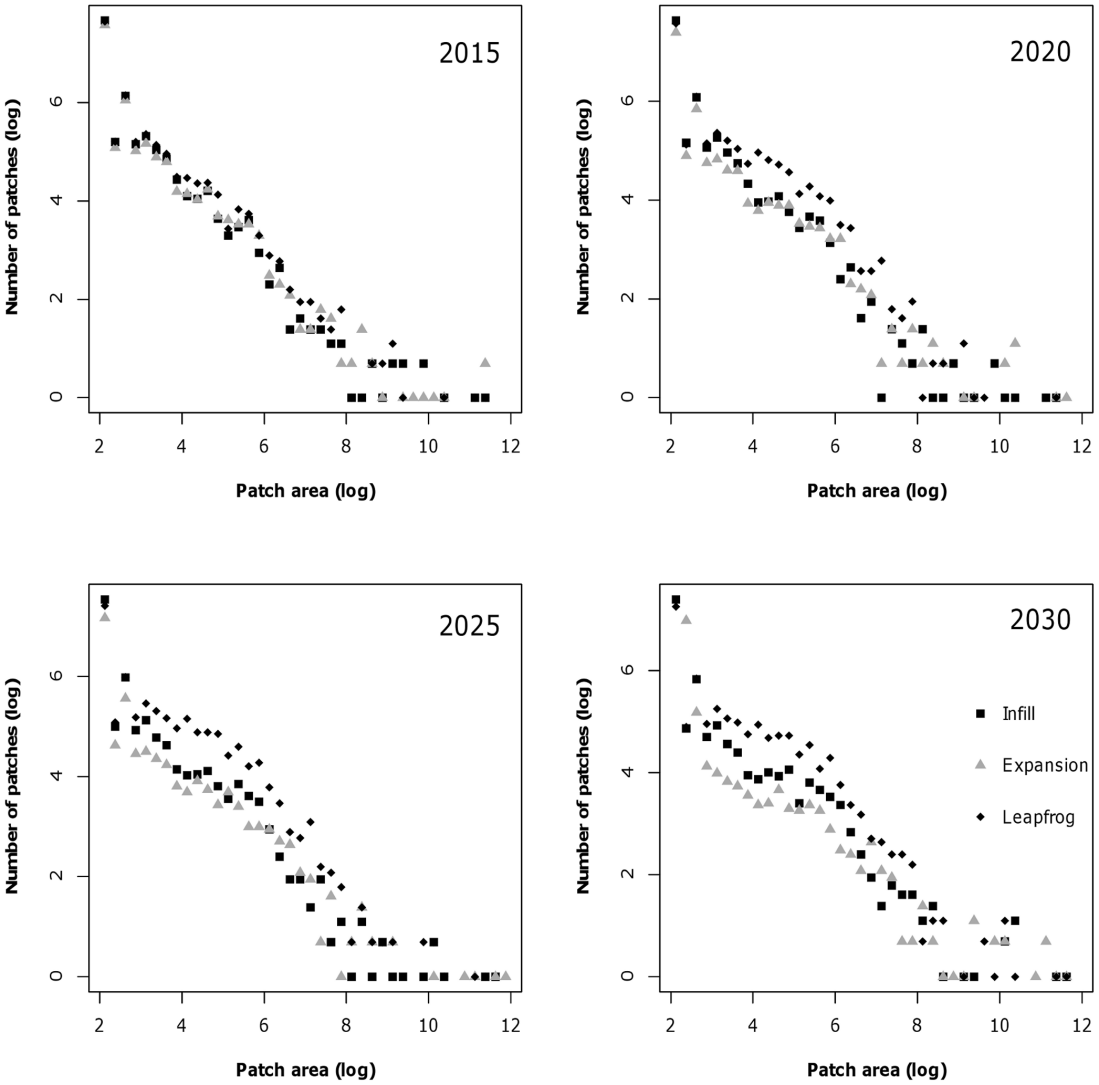
Trends in number of patches and patch area for three planning policy simulations from 2010–2030. Numbers were log-transformed for better visual representation of the scenarios.

The land use simulation model, based on a combination of the econometric subdivision model and three different growth policies, resulted in substantial differences in the extent and pattern of housing of the three scenarios. The total area of housing development, or the housing footprint, was largest for simulations where leapfrog growth dominated, followed by expansion-type development, and then infill (Figure 1a). The differences in the housing footprint became larger among the scenarios over time, but the largest difference was between infill and the other two development types. As the housing footprint expanded in the three scenarios, the corresponding housing density declined, so that leapfrog growth resulted in the lowest housing density per 1-km, followed by expansion and then infill (Figure 2b). Despite the near inverse of this relationship, there was generally a larger separation among scenarios with regard to housing density. With larger housing footprints and lower housing density, the number of separate housing clusters increased while their size decreased (Figure 2c).

In the first two time steps of the model (2015 and 2020), the simulated development pattern closely followed the desired pattern in the scenario, although some of the growth in the infill scenario ended up becoming expansion or leapfrog (Table 2). In the last two time steps (2025 and 2030), there were not enough infill parcels left, and thus, the majority of growth in these simulations became expansion, followed by infill, and then leapfrog. In the last time step, there were not enough isolated parcels in the leapfrog scenario and thus, the majority of development became expansion. Thus in general, as more development occurred in the simulations by the year 2030, the majority took the form of expansion.

**Table 2:** Pattern of simulated development under infill, expansion, and leapfrog growth policies.

		Actual development		
Development scenario	year	*Infill*	*Expansion*	*Leapfrog*
*Infill*	2015	9450	18	6
	2020	11787	153	29
	2025	236	624	144
	2030	325	890	179
*Expansion*	2015	0	772	0
	2020	0	1243	2
	2025	0	1871	1
	2030	0	2662	0
*Leapfrog*	2015	0	10	408
	2020	0	5	1132
	2025	1	83	3563
	2030	34	917	0

The numbers in the table denote the numbers of patches of a given development type.

**Figure 3:**
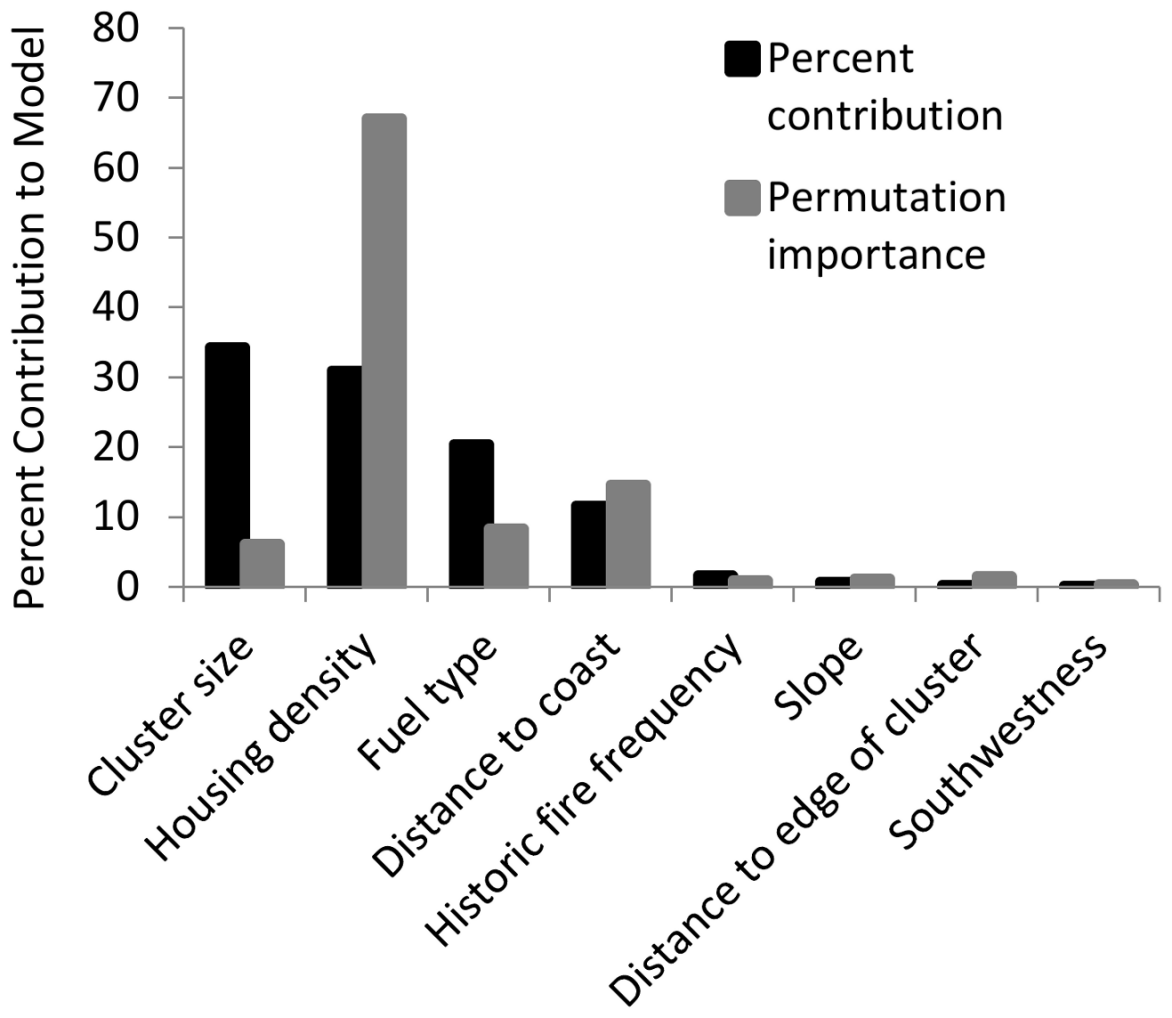
The importance of explanatory variables averaged across five cross-validated replications in the MaxEnt fire risk model. Percent contribution is determined as a function of the information gain from each environmental variable throughout the MaxEnt model iterations. Permutation importance reflects the drop in model accuracy that results from random permutations of each environmental variable, normalized to percentages.

The area under the curve (AUC) of receiver operating characteristic (ROC) plots, indicating the ability of the MaxEnt model to discriminate between burned and unburned structures, averaged across five cross-validated replicate runs was 0.91. The AUC represents the probability that, for a randomly selected set of observations, the model prediction was higher for a burned structure than for an unburned structure [49].The two most important variables in the model according to the internal jackknife tests in MaxEnt [47] were related to housing pattern: low to intermediate housing density and small cluster size and housing density (Figure 3). The distance to the edge of housing cluster was a less important contribution.

**Figure 4:**
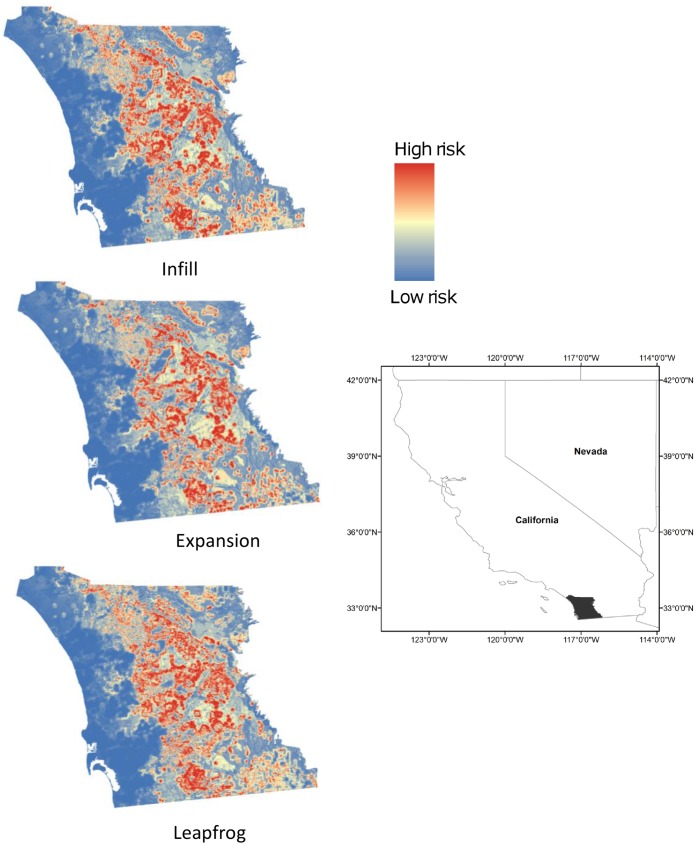
Maps of the study area showing projected wildfire risk at year 2030 for simulations of residential development under policies emphasizing infill, expansion, or leapfrog growth.

Maps showing the probability of a structure being destroyed in a wildfire, displayed as a gradient from low to high risk, show broad agreement relative to the general areas of the landscape that are riskiest, with correlation coefficients ranging from 0.85–0.91 (Figure 4). Nevertheless, subtle differences are apparent in the three development-scenario maps by year 2030, with the highest-risk areas in the expansion scenario located farther east than infill, and the highest-risk areas in leapfrog occupying a wider extent than either of the other two scenarios.

**Figure 5:**
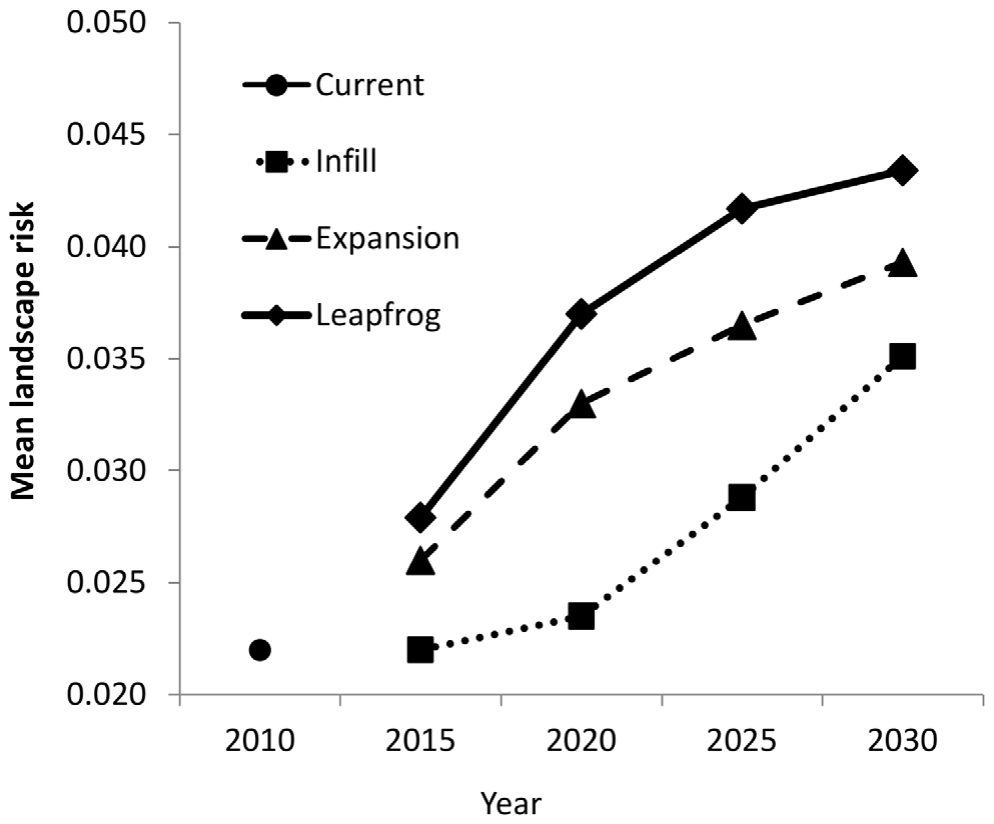
Projected landscape fire risk, reflecting the probability of burning in a wildfire averaged across all residential structures on the current landscape and in three development scenarios of infill, expansion, and leapfrog for year 2030.

Differences among current housing and the three development scenarios are clearly illustrated through the mean landscape risk, or total probability of all structures burning (Figure 5). All three development scenarios were predicted to experience an increase in mean landscape risk over the duration of the simulations, except for infill at year 2015. The highest landscape risk to structures was predicted for the leapfrog scenario, followed by expansion, and then infill. The increase in risk over time is more gradual for the infill scenario than the other two scenarios.

**Figure 6:**
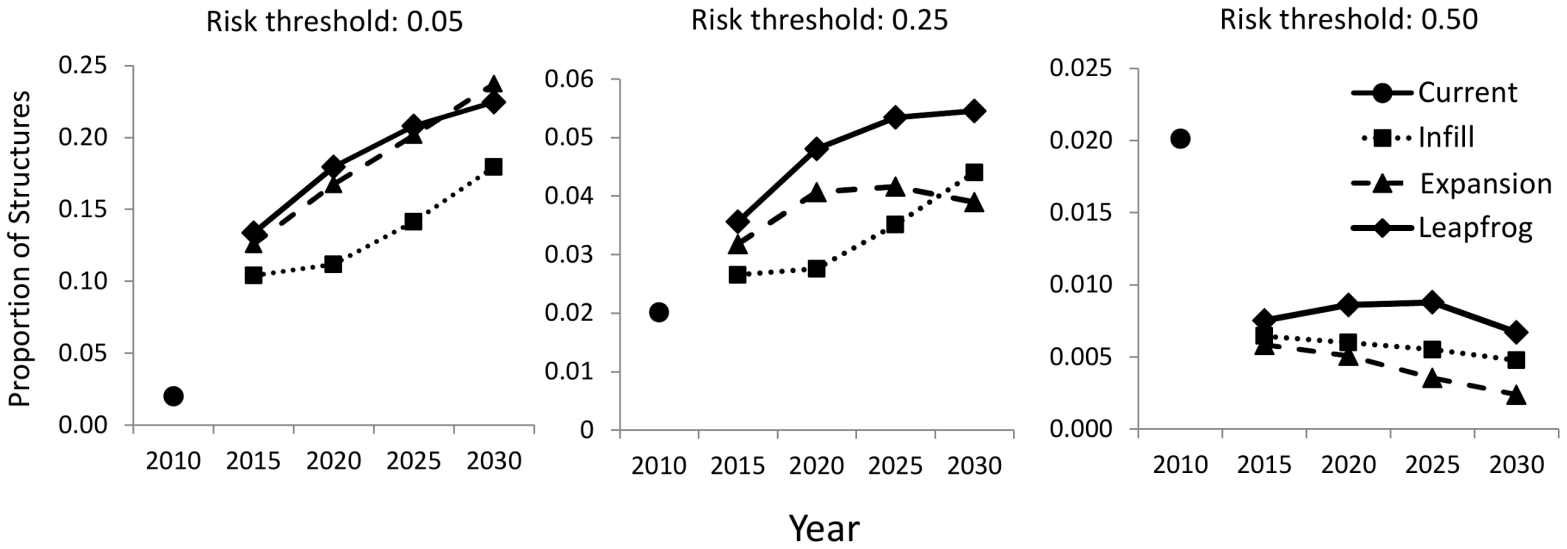
Proportion of residential structures that are located in areas of high fire risk defined using thresholds from the fire risk model of 0.05, 0.25, and 0.5 for current structures and for structures simulated under infill, expansion, and leapfrog growth policies.

The ranking of scenarios varied according to the proportion of structures located within different levels of risk defined through binary thresholding (Figure 6). When the continuous risk maps were thresholded at the lowest number of 0.05, a large proportion of structures in all scenarios fell within areas defined as risky according to this criterion. At this threshold, the proportion of structures in high-risk areas increased linearly for the expansion and leapfrog development scenarios while the proportion of infill homes increased more gradually. When risk was defined more conservatively at 0.25, temporal trends for the leapfrog and infill scenarios were similar to the 0.05 threshold. However, the proportion of structures at risk in the expansion scenario initially increased to 2020, but this proportion leveled off and declined by 2030. When the threshold was highest at 0.50, a very low proportion of structures in any scenario were located in areas at risk. But in these high-risk areas, the expansion scenario switched places with infill to have the lowest proportion of structures at risk in all time steps. Leapfrog had the largest proportion of homes at risk. This proportion of homes located in areas at risk with a threshold at 0.5 declined over time for all three scenarios.

## Discussion

Our simulations of residential development showed that planning policies based on different growth types, applied locally for subdivision of individual parcels, will likely produce substantial and cumulative landscape-level differences in pattern, location, and extent of development. These differences in development pattern, in turn, will likely affect the area and proportion of structures at risk from burning in wildfires. In particular, the scenarios with lower housing density and larger numbers of small, isolated clusters of development, i.e., leapfrog followed by expansion and infill, were generally predicted to have the highest predicted fire risk to the largest proportion of structures in the study area. Nevertheless, rankings of scenarios were affected by the definition of risk.

Theoretically, it makes sense that leapfrog development produced fragmented development with larger numbers of small patches, lower housing density, and a larger housing footprint; and that infill resulted in the opposite, with expansion in the middle. By definition, leapfrog development requires open space around all sides of the newly developed parcel, whereas infill requires development on all sides, and expansion requires development on one side and open space on another. Implementing these planning policies on real landscapes, however, can be complex if there are more houses to build than there are parcels that meet the definitions of the three planning rules, and thus not all development conforms strictly to the policy [54]. In our simulations, parcels meeting the definition of each growth type had a higher probability of subdividing; yet, as we were simulating a real landscape, many newly developed parcels did not meet the scenario criteria. That the three scenarios nevertheless produced substantial differences in landscape-level development patterns shows that decision-making at the individual level can lead to meaningful broad-scale effects.

The objective of the econometric model was to provide a baseline probability to predict which parcels were most likely to subdivide; thus, the econometric model itself provides no explanation of how a given policy affects likelihood of subdivision, although it does indicate the correlation between the policy and the outcome. In our setting, which areas are protected, marked for redevelopment, or marked for development may be endogenous to the land owner decision to subdivide. In the case of these variables especially, our results should not be interpreted as causal predictors. Likewise, we use data only from 2005–2009 to predict changes to 2030. If major changes in the land market take place over this time horizon our model will not be able to take this into account.

Although some differences in predicted fire risk among the three scenarios likely stemmed from location of new structures relative to variables such as distance to coast, fuel type, or slope, the most important variables in the fire risk model were housing density and cluster size, with most structure loss historically occurring in areas with low housing density and in small, isolated housing clusters. Thus, leapfrog development was generally the riskiest scenario and infill the least risky. The most surprising result was the variation in predicted risk for the expansion scenario over time and at different thresholds. While leapfrog and infill showed similar trajectories across thresholds, expansion went from being the highest-risk scenario at the low threshold to being the lowest-risk scenario at the highest threshold. Because the threshold is merely a way to group structures into a binary classification, this means that, while the average risk calculated across all homes shows expansion to rank in the middle of infill and leapfrog throughout the simulation (Figure 5), the other two scenarios have a relatively larger proportion of homes that are modeled to be at a very high risk (i.e., 0.25 or 0.5), particularly by the end of the simulations. Because the total number of structures with a risk greater than 0.25 or 0.5 is relatively low in all scenarios, this difference in distribution of homes at the highest risk is not reflected in the mean. Another reason for the shift in rank of expansion over time is that, as more development occupied the landscape, there were fewer parcels remaining to accomplish infill or leapfrog type growth in the other scenarios. Thus, by the end of the simulations in year 2030, the majority of growth in all scenarios was expansion, and there was some convergence between scenarios. Finally, the change in risk of expansion growth over time may reflect that, despite the relatively low importance of distance to edge of cluster as an explanatory variable, expansion growth is characterized as having an initially fragmented landscape pattern that eventually merges into large patches with low edge.

Although leapfrog development clearly ranked highest in terms of fire risk, the interpretation of which planning policy is best may depend on fire management objectives and resources, as well as other considerations such as biodiversity or ecological impacts. The spatial pattern of development affects multiple ecological functions and services [55], with potentially varying conservation implications; both leapfrog and expansion development consumed more land than infill, which would likely lead to more ecological degradation [56]; nevertheless, higher-density clustered development may be dominated by more invasive species [57]. Trade-offs between fire protection and conservation are common, but techniques are available for identifying mutually beneficial solutions [58].

Different perceptions of the fire risk results could also potentially translate into different planning priorities for management. For example, if the priority is to plan for the lowest overall risk to structures, then the mean landscape risk clearly delineates the rankings of options, with infill being the winner. However, if the objective is to reduce the number of structures at the highest risk threshold, i.e., > = 0.5, then expansion is the best option, at least by 2030. An important consideration for fire management is the total area that needs to be protected, as well as the length of wildland-urban interface [8], [13]. Therefore, despite the lower number of structures at the highest risk thresholds, expansion creates more edge than infill and may translate into greater challenges for firefighter protection.

Although we did not create separate scenarios for high or low growth, the results at different time steps can be substituted to envision the potential outcome of developing more or fewer houses. In the short term, the total fire risk is projected to increase proportionately as more land is developed. However, given the inverse relationship between housing density and fire risk, it is possible that this trend could reverse if housing growth eventually resulted in expansive high-density development.

Land use planning is one of a range of options available for reducing fire risk, and the best outcome will likely be achieved through a combination of strategies that include homeowner actions, improvements in fire-safe building codes, and advanced fire suppression tactics. Although we isolated the effect of land use planning policy in the three development scenarios, the fire risk model nevertheless showed that the pattern and location of structures in this study area were the most important out of a suite of factors influencing structure loss. We used a correlative approach that did not incorporate mechanisms or feedbacks, but our models clearly illustrated differences in the cumulative effects of individual planning decisions. The relationship between spatial pattern of development and fire risk is likely related to the intermixing of development and wildland vegetation [29], [59]; thus, these results likely apply to a wide range of fire-prone ecosystems with large proportions of human-caused ignitions. Nevertheless, because fire risk is highly variable over space and time, and due to a range of human and biophysical variables [60], we recommend planners develop their own models for the best understanding of where the most fire-prone areas are in their region [19].

With projections of substantial global change in climate and human development, we recommend that land use planning should be considered as an important component to fire risk management, potentially to become as successful as the prevention of building on flood plains [61]. History has shown us that preventing fires is impossible in areas where large wildfires are a natural ecological process [4], [9]. As Roger Kennedy put it, “the problem isn’t fires; the problem is people in the wrong places [62].”

## Supporting Information

Table S1 **Definitions and summary statistics for variables used in the probit model.**
(DOCX)
