# Supplementary material for: Land Use Planning and Wildfire: Development Policies Influence Future Probability of Housing Loss
Source: PLoS One. 2013 Aug 14;8(8):e71708. doi: 10.1371/journal.pone.0071708 (PMC3743760; doi:10.1371/journal.pone.0071708)
Supplement: Table S1 — Definitions and summary statistics for variables used in the probit model. (DOCX) [file pone.0071708.s001.docx]

Table S1. Definitions and summary statistics for variables used in the probit model.

| Variable class | Variable | Mean | Std. Dev | Min | Max |
| --- | --- | --- | --- | --- | --- |
| Dependent Variable | **Subdivided (1=yes,0=no)** | 0.005045 | 0.070847 | 0 | 1 |
| Parcel Size Characteristics | **Acres of lot** | 11.53121 | 46.9977 | 0.250003 | 712.2959 |
|  | **Acres of lot ^2^** | 2341.732 | 22537.22 | 0.062502 | 507365.4 |
|  | **Parcel between 10 and 20 acres (1=yes,0=no)** | 0.053532 | 0.225092 | 0 | 1 |
|  | **Parcel between 5-10 acres (1=yes,0=no)** | 0.096443 | 0.295199 | 0 | 1 |
|  | **Parcel between 2-5 acres (1=yes,0=no)** | 0.257029 | 0.436998 | 0 | 1 |
|  | **Parcel between 1-2 acres (1=yes,0=no)** | 0.09322 | 0.290742 | 0 | 1 |
|  | **Parcel between .5-1 acres (1=yes,0=no)** | 0.181991 | 0.385839 | 0 | 1 |
|  | **Parcel between .25-.5 acres (1=yes,0=no)** | 0.226157 | 0.418344 | 0 | 1 |
| Parcel Location Characteristics | **Distance to ocean** | 70766.83 | 64935.94 | 0 | 356528 |
|  | **Distance to ocean ^2^** | 9.22E+09 | 1.75E+10 | 0 | 1.27E+11 |
|  | **Distance to major road** | 11625.64 | 26850.55 | 0 | 209797.5 |
|  | **Distance to major road ^2^** | 8.56E+08 | 3.93E+09 | 0 | 4.40E+10 |
|  | **Distance to nearest city center** | 33953.54 | 40814.13 | 0 | 258506.1 |
|  | **Distance to nearest city center ^2^** | 2.82E+09 | 7.89E+09 | 0 | 6.68E+10 |
| Parcel slope characteristics | **Slope between 0-5%** | 0.494768 | 0.499975 | 0 | 1 |
|  | **Slope between 5-10%** | 0.357226 | 0.479184 | 0 | 1 |
|  | **Slope between 10-25%** | 0.141946 | 0.348996 | 0 | 1 |
| Land Use/Zoning | **Rural Residential – zone rural residential (1=yes,0=no)** | 0.204349 | 0.403227 | 0 | 1 |
|  | **Single Family – zoned single family (1=yes,0=no)** | 0.316283 | 0.465027 | 0 | 1 |
|  | **Multi-Family – zoned multifamily (1=yes,0=no)** | 0.032392 | 0.177039 | 0 | 1 |
|  | **Open Space – open space land use (1=yes,0=no)** | 0.134081 | 0.340741 | 0 | 1 |
|  | **Orchard/Vineyard – zoned agricultural - orchard vineyard land use (1=yes,0=no)** | 0.036869 | 0.188441 | 0 | 1 |
|  | **Agriculture – zoned agricultural non orchard or vineyard land use (1=yes,0=no)** | 0.029133 | 0.168179 | 0 | 1 |
|  | **Vacant Land – vacant lot land use (1=yes,0=no)** | 0.014484 | 0.119475 | 0 | 1 |
|  | **Zoned protected - zoned as a protected area (1=yes,0=no)** | 0.156301 | 0.363142 | 0 | 1 |
| Special development areas | **Area marked for redevelopment** | 0.034397 | 0.182247 | 0 | 1 |
|  | **Area marked for development** | 0.02634 | 0.160145 | 0 | 1 |
